# Supplementary material for: Development of an intraductal papillary mucinous neoplasm malignancy prediction scoring system
Source: PLoS One. 2024 Oct 17;19(10):e0312234. doi: 10.1371/journal.pone.0312234 (PMC11486388; doi:10.1371/journal.pone.0312234)
Supplement: S1 Table — (DOCX) [file pone.0312234.s001.docx]

| Sp Table 1. Distribution of IMAP Score and Malignancy | | | |
| --- | --- | --- | --- |
|  | Benign | Malignant | Total |
| IMAP score | N (%) | N (%) | N (%) |
| 0 | 13 (14.6) | 1 (1.4) | 14 (8.75) |
| 1 | 20 (22.5) | 3 (4.2) | 23 (14.4) |
| 2 | 29 (32.6) | 11 (15.5) | 40 (25.0) |
| 3 | 15 (16.9) | 20 (28.2) | 35 (21.9) |
| 4 | 12 (13.5) | 15 (21.1) | 27 (16.9) |
| 5 | 0 (0.0) | 6 (8.5) | 6 (3.8) |
| 6 | 0 (0.0) | 7 (9.9) | 7 (4.4) |
| 7 | 0 (0.0) | 5 (7.0) | 5 (3.1) |
| 8 | 0 (0.0) | 1 (1.4) | 1 (0.6) |
| 9 | 0 (0.0) | 2 (2.8) | 2 (1.3) |
| Total | 89 (100) | 71 (100) | 160 (100) |
| IMAP, IPMN malignancy prediction; IPMN, intraductal papillary mucinous neoplasm | | | |
