# Supplementary material for: Development of an intraductal papillary mucinous neoplasm malignancy prediction scoring system
Source: PLoS One. 2024 Oct 17;19(10):e0312234. doi: 10.1371/journal.pone.0312234 (PMC11486388; doi:10.1371/journal.pone.0312234)
Supplement: S2 Table — (DOCX) [file pone.0312234.s002.docx]

| Sp Table 2. Characteristics of the IPMN patients in the validation cases | | | | |
| --- | --- | --- | --- | --- |
|  |  | Benign | Malignant | p |
| Total number | N (%) | 16 (72.7) | 6 (27.3) |  |
| Age (years) | Median (IQR) | 74 (67-77.5) | 74 (71-74) | 0.970 |
| <65 years | N (%) | 3 (18.8) | 0 (0.0) | 0.254 |
| >65 years | N (%) | 13 (81.3) | 6 (100) |  |
| Sex |  |  |  |  |
| Male | N (%) | 12 (75.0) | 5 (83.3) | 0.678 |
| Female | N (%) | 4 (25.0) | 1 (16.7) |  |
| Type of IPMN |  |  |  |  |
| Branch duct type | N (%) | 5 (31.3) | 2 (33.3) | **0.044** |
| Mixed type | N (%) | 11 (68.8) | 2 (33.3) |  |
| Main duct type | N (%) | 0 (0.0) | 2 (33.3) |  |
| Site of lesion |  |  |  |  |
| Head | N (%) | 11 (68.8) | 5 (83.3) | 0.641 |
| Body | N (%) | 2 (12.5) | 0 (0.0) |  |
| Tail | N (%) | 3 (18.8) | 1 (16.7) |  |
| Pancreatitis |  |  |  |  |
| absent | N (%) | 15 (93.8) | 6 (100) | 0.531 |
| present | N (%) | 1 (6.3) | 0 (0.0) |  |
| Diabetes mellitus |  |  |  |  |
| absent | N (%) | 13 (81.3) | 4 (66.7) | 0.467 |
| present | N (%) | 3 (18.8) | 2 (33.3) |  |
| new onset / rapid exacerbation | N (%) | 0 (0.0) | 0 (0.0) |  |
| Jaundice |  |  |  |  |
| absent | N (%) | 16 (100) | 6 (100) |  |
| present | N (%) | 0 (0.0) | 0 (0.0) |  |
| CEA (ng/ml) | Median (IQR) | 2.15 (1.6-3.5) | 2.75 (1.9-4.1) | 0.507 |
| <5 ng/ml | N (%) | 14 (87.5) | 5 (83.3) | 0.800 |
| >5 ng/ml | N (%) | 2 (12.5) | 1 (16.7) |  |
| CA19-9 (U/ml) | Median (IQR) | 12.25 (5.85-25.4) | 23.5 (7.8-32.5) | 0.439 |
| <37 U/ml | N (%) | 15 (93.8) | 5 (83.3) | 0.449 |
| >37 U/ml | N (%) | 1 (6.3) | 1 (16.7) |  |
| CT |  |  |  |  |
| absent | N (%) | 0 (0.0) | 0 (0.0) | 0.837 |
| present (without contrast) | N (%) | 0 (0.0) | 1 (16.7) |  |
| present (with contrast) | N (%) | 16 (100) | 5 (83.3) |  |
| Time to surgery from CT (days) | Median (IQR) | 50.5 (17.5-94.5) | 63.5 (40-83) | 0.912 |
| MRI |  |  |  |  |
| absent | N (%) | 0 (0.0) | 0 (0.0) | 0.910 |
| present (without contrast) | N (%) | 13 (81.3) | 5 (83.3) |  |
| present (with contrast) | N (%) | 3 (18.8) | 1 (16.7) |  |
| Time to surgery from MRI (days) | Median (IQR) | 94 (50-153) | 116.5 (85-233) | 0.338 |
| EUS |  |  |  |  |
| absent | N (%) | 0 (0.0) | 0 (0.0) | **0.032** |
| present (without contrast) | N (%) | 13 (81.3) | 2 (33.3) |  |
| present (with contrast) | N (%) | 3 (18.8) | 4 (66.7) |  |
| Time to surgery from EUS (days) | Median (IQR) | 104 (69-213.5) | 100.5 (57-184) | 0.606 |
| Pathology |  |  |  |  |
| Low-grade dysplasia | N (%) | 16 (100) | 0 (0.0) | **<0.001** |
| High-grade dysplasia | N (%) | 0 (0.0) | 3 (50.0) |  |
| IPMC | N (%) | 0 (0.0) | 3 (50.0) |  |
| Statistically significant p-values are indicated in bold. | | | | |
| IPMN, intraductal papillary mucinous neoplasm; IQR, interquartile range; CEA, carcinoembryonic antigen; CA19-9, carbohydrate antigen 19-9; CT, computed tomography; MRI, magnetic resonance imaging; EUS, endoscopic ultrasonography; IPMC, intraductal papillary mucinous carcinoma | | | | |
