# Supplementary material for: Development of an intraductal papillary mucinous neoplasm malignancy prediction scoring system
Source: PLoS One. 2024 Oct 17;19(10):e0312234. doi: 10.1371/journal.pone.0312234 (PMC11486388; doi:10.1371/journal.pone.0312234)
Supplement: S3 Table — (DOCX) [file pone.0312234.s003.docx]

| Sp Table 3. Distribution of IMAP Score and Malignancy in the validation cases | | | |
| --- | --- | --- | --- |
|  | Benign | Malignant | Total |
| IMAP score | N (%) | N (%) | N (%) |
| 0 | 0 (0.0) | 0 (0.0) | 0 (0.0) |
| 1 | 1 (6.3) | 0 (0.0) | 1 (4.6) |
| 2 | 5 (31.3) | 1 (16.7) | 6 (27.3) |
| 3 | 7 (43.8) | 2 (33.3) | 9 (40.9) |
| 4 | 2 (12.5) | 1 (16.7) | 3 (13.6) |
| 5 | 1 (6.3) | 1 (16.7) | 2 (9.1) |
| 6 | 0 (0.0) | 0 (0.0) | 0 (0.0) |
| 7 | 0 (0.0) | 0 (0.0) | 0 (0.0) |
| 8 | 0 (0.0) | 1 (16.7) | 1 (4.6) |
| 9 | 0 (0.0) | 0 (0.0) | 0 (0.0) |
| Total | 16 (100) | 6 (100) | 22 (100) |
| IMAP, IPMN malignancy prediction; IPMN, intraductal papillary mucinous neoplasm | | | |
